# Biochemical elucidation of citrate accumulation in *Synechocystis* sp. PCC 6803 via kinetic analysis of aconitase

**DOI:** 10.1038/s41598-021-96432-2

**Published:** 2021-08-24

**Authors:** Maki Nishii, Shoki Ito, Noriaki Katayama, Takashi Osanai

**Affiliations:** School of Agriculture, Meiji University, 1-1-1 Higashimita, Tama-ku, Kawasaki, Kanagawa 214-8571 Japan

**Keywords:** Enzymes, Biochemistry, Microbiology

## Abstract

A unicellular cyanobacterium *Synechocystis* sp. PCC 6803 possesses a unique tricarboxylic acid (TCA) cycle, wherein the intracellular citrate levels are approximately 1.5–10 times higher than the levels of other TCA cycle metabolite. Aconitase catalyses the reversible isomerisation of citrate and isocitrate. Herein, we biochemically analysed *Synechocystis* sp. PCC 6803 aconitase (*Sy*AcnB), using citrate and isocitrate as the substrates. We observed that the activity of *Sy*AcnB for citrate was highest at pH 7.7 and 45 °C and for isocitrate at pH 8.0 and 53 °C. The *K*_m_ value of *Sy*AcnB for citrate was higher than that for isocitrate under the same conditions. The *K*_m_ value of *Sy*AcnB for isocitrate was 3.6-fold higher than the reported *K*_m_ values of isocitrate dehydrogenase for isocitrate. Therefore, we suggest that citrate accumulation depends on the enzyme kinetics of *Sy*AcnB, and 2-oxoglutarate production depends on the chemical equilibrium in this cyanobacterium.

## Introduction

Cyanobacteria are bacteria that can perform oxygenic photosynthesis and produce a variety of metabolites from carbon dioxide. *Synechocystis* sp. PCC 6803 (*Synechocystis* 6803) is a well-studied model cyanobacterium, as its genome has been sequenced^1^ and it can be easily transformed and has the ability to multiply rapidly.

The tricarboxylic acid (TCA) cycle is one of the most important bacterial metabolic pathways. The oxidative TCA cycle produces 2-oxoglutarate (2-OG), a precursor for amino acid production, from oxaloacetate, citrate, and isocitrate^2,3^. Aconitase (EC 4.2.1.3) is the enzyme that catalyses the second reaction in the TCA cycle, i.e., it reversibly isomerises citrate and isocitrate via *cis*-aconitate^4^. This enzyme is encoded by the *acnB* gene and contains a [4Fe-4S] cluster. Bacterial aconitase is a bifunctional protein, and it binds to mRNA when the Fe-S cluster is disrupted by lack of iron and oxidative stress, thereby regulating gene expression^5,6^. In the cells of *Synechocystis* 6803, Fe-S clusters are generated by Suf proteins and inserted into apo-proteins^7,8^. There are two genetically distinct aconitases in bacteria. *Escherichia coli* possesses two aconitases, namely, aconitase A (AcnA) and aconitase B (AcnB); AcnB is unstable under in vitro conditions^9^. The amino acid sequences of the two enzymes are approximately 17% identical^10^. In *E. coli*, AcnB is the major enzyme of the TCA cycle and is synthesised during the exponential growth phase, whereas AcnA is expressed during the stationary phase under conditions of iron deficiency and oxidative stress^11,12^.

The genes involved in the oxidative TCA cycle in cyanobacteria are essential^13^, and the cyanobacterial TCA cycle was thought to be incomplete, as cyanobacteria lack 2-oxoglutarate dehydrogenase. *Synechocystis* 6803 can convert 2-OG to succinate by two alternative pathways. The first pathway involves two enzymes, namely, 2-OG decarboxylase and succinic semialdehyde dehydrogenase^14,15^, and the second pathway is the γ-aminobutyric acid shunt pathway^16^. Intracellular citrate levels in *Synechocystis* 6803 are 10-fold higher than malate, fumarate, succinate, and 2-OG levels and 1.5-fold higher than the isocitrate levels^17^. These results suggest that citrate functions as a pool of carbon source in this cyanobacterium. Citrate also plays a key role in the regulation of sugar metabolism in *Synechocystis* 6803 because it specifically inhibits the enzymes of the oxidative pentose phosphate pathway, namely, glucose-6-phosphate dehydrogenase and 6-phosphogluconate dehydrogenase^18^. Additionally, the expression and abundance of *Synechocystis* 6803 aconitase (*Sy*AcnB) vary according to culture conditions. *Sy*AcnB abundance increases by 3.8-fold following 48 h of nitrogen depletion, compared to that under photoautotrophic conditions^19^. Furthermore, the *acnB* transcript levels increase by more than 2-fold of the original level after 2 h of nitrogen depletion in *Synechocystis* 6803^20^. These results indicate that it is important for *Synechocystis* 6803 to regulate the citrate level and its related enzyme aconitase to adapt to environmental changes.

However, limited information is available about the biochemical properties of aconitase in bacteria containing only AcnB. The *V*_max_ and *K*_m_ values of *Sy*AcnB for *cis*-aconitate have been determined^21^, but the biochemical characteristics of *Sy*AcnB using citrate and isocitrate as substrates have not been investigated. In this study, we determined the optimal conditions, kinetic parameters, and the influence of other TCA metabolites on *Sy*AcnB using citrate and isocitrate as substrates. Overall, our biochemical analyses elucidated the metabolic flow of citrate in *Synechocystis* 6803.

## Results

### Purification and reactivation conditions of *Sy*AcnB

To determine whether aconitase is the only enzyme in *Synechocystis* 6803 that uses citrate as a substrate, BLAST search was performed. *Synechocystis* 6803 did not possess genes encoding ATP-citrate lyase (ACL) and citryl-CoA synthase/citryl-CoA lyase (CCS/CCL) which cleave citrate (Table 1). The results of the BLAST search showed that *Synechocystis* 6803 possesses only AcnB (Table S1).

**Table 1:** BLAST search results for ATP-citrate lyase, citryl-CoA synthetase, and citryl-CoA lyase.

Query sequence	CyanoBase ID	Name	K number	Bits	E-value
A (ATP-citrate lyase)	sll1557	*sucD*; succinyl-CoA synthetase	K01902	68.2	2e–13
	sll0401	*gltA*; citrate synthase	K01647	50.8	1e-07
	slr1495	Unknown protein		30.4	0.38
	sll0283	Unknown protein		28.1	1.7
	sll1174	Unknown protein		27.3	2.4
	sll1556	Hypothetical protein	K01823	26.2	5.7
	sll1858	Unknown protein		26.2	6.7
	slr0220	*glyS*; glycyl-tRNA synthetase beta chain	K01879	26.2	8.1
	sll1406	*fhuA*; ferrichrome-iron receptor	K02014	25.8	8.4
	sll2001	*lap*; leucine aminopeptidase	K01255	25.8	9.2
B (ATP-citrate lyase)	sll1023	*sucC*; succinate-CoA ligase	K01903	50.4	7e–08
	slr1661	Unknown protein	K07326	27.3	1.7
	sll1959	*suhB*; extragenic suppressor		25.4	5.5
	sll1291	PatA subfamily	K02657	25.4	7.5
	slr1855	Unknown protein		25.4	8.0
	slr2104	Hybrid sensory kinase	K11527	25.4	8.4
	slr0301	*ppsA*; phosphoenolpyruvate synthase	K01007	25.4	8.4
C (citryl-CoA synthetase)	sll1557	*sucD*; succinyl-CoA synthetase	K01902	94.4	4e–23
	slr0058	Unknown protein		25.8	3.1
	sll0816	Hypothetical protein		26.2	3.4
	sll1687	PleD gene product homologue		25.8	4.5
	slr2124	Short-chain alcohol dehydrogenase family		25.4	5.5
	sll0361	Unknown protein		25.0	5.6
	sll8034	2-nitropropane dioxygenase		25.4	6.1
	slr1434	*pntB*; pyridine nucleotide transhydrogenase beta subunit	K00325	25.4	6.6
	sll0726	*pgm*; phosphoglucomutase	K01835	25.0	7.6
	sll0833	OppC in a binding protein-dependent transport system	K02034	24.6	8.9
D (citryl-CoA synthetase)	sll1023	*sucC*; succinate–CoA ligase	K01903	66.6	6e–13
	slr0213	*guaA*; GMP synthetase	K01951	28.1	1.3
	sll5076	Hypothetical protein		26.9	1.6
	sll1632	Unknown protein	K03589	26.2	3.5
	sll1099	*tufA*; protein synthesis elongation factor Tu	K02358	25.0	8.9
E (citryl-CoA lyase)	sll0401	*gltA*; citrate synthase	K01647	68.2	4e–14
	slr0733	*xerC*; integrase-recombinase protein	K03733	27.7	0.65
	sll0265	Unknown protein		25.8	2.5
	slr0105	Unknown protein		25.4	3.6
	slr1350	*desA*; fatty acid desaturase	K10255	25.4	3.9
	sll0622	*nadA*; quinolinate synthetase	K03517	25.0	4.8
	slr1411	Unknown protein	K09121	25.0	5.2
	slr1462	Unknown protein	K06883	24.6	6.5
	sll8019	Hypothetical protein		24.6	7.9
	sll0757	*purF*; amidophosphoribosyltransferase	K00764	24.3	9.6

BLAST search for ATP-citrate lyase, citryl-CoA synthetase, and citryl-CoA lyase was performed using the Kyoto Encyclopedia of Genes and Genomes database (https://www.genome.jp/kegg/genome.html). The following sequences were used for the search: A: ATP-citrate lyase alpha-subunit from *Chlorobium limicola* (Clim_1231), B: ATP-citrate lyase beta-subunit from *Chlorobium limicola* (Clim_1232), C: citryl-CoA synthetase small subunit from *Hydrogenobacter thermophilus* (HTH_0201), D: citryl-CoA synthetase large subunit from *Hydrogenobacter thermophilus* (HTH_1737), E: citryl-CoA lyase from *Hydrogenobacter thermophilus* (HTH_0311).

**Figure 1:**
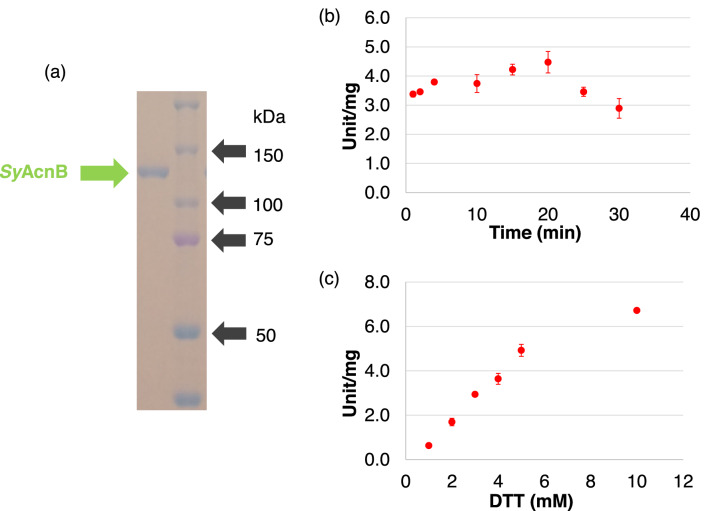
Determination of reactivation conditions for *Synechocystis* sp. PCC 6803 aconitase B (*Sy*AcnB). (**a**) Affinity purification results for GST-tagged *Sy*AcnB. The purified protein was electrophoresed via 8% SDS-PAGE, and the gel was stained using Instant Blue reagent. (**b**) Effect of reactivation time on *Sy*AcnB activity. The experiment was performed using 50 pmol of *Sy*AcnB and 20 mM trisodium citrate dihydrate in Tris–HCl buffer pH 8.0 at 45 °C. Mean ± SD values were calculated from three independent experiments. (**c**) Effect of DTT concentration on *Sy*AcnB activity. The experiment was performed using 50 pmol of *Sy*AcnB and 20 mM trisodium citrate dihydrate in Tris–HCl buffer pH 7.7 at 45 °C. Mean ± SD values were calculated from three independent experiments. GST, glutathione-*S*-transferase; SDS-PAGE, sodium dodecyl sulphate–polyacrylamide gel electrophoresis; DTT, dl-dithiothreitol; SD, standard deviation.

We expressed the GST-tagged *Sy*AcnB in *E. coli* DH5α and purified it using affinity chromatography (Fig. 1a). No *Sy*AcnB activity was observed in the apoenzyme state, (without reactivation). The activity of *Sy*AcnB with citrate as the substrate was 76% of its maximum activity at 1 min after the addition of the reagents; the activity peaked at 20 min and then gradually decreased (Fig. 1b). The activity of *Sy*AcnB for citrate increased depending on the concentration of the reducing agent DTT (1–5 mM) added for enzyme reactivation (Fig. 1c). Hereafter, the reactivation of *Sy*AcnB was carried out with 5 mM DTT for 20 min, similar to a previous study^22^. Na_2_S and (NH_4_)_2_Fe(SO_4_)_2_·6H_2_O were added to the mixture after the addition of DTT.

### Kinetic parameters of *Sy*AcnB

**Figure 2:**
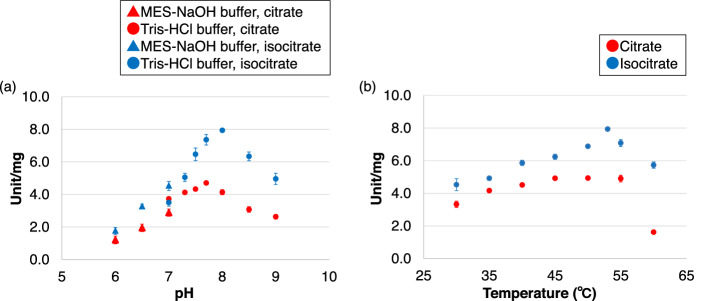
Optimal pH and temperature for *Sy*AcnB. (**a**) Effect of pH on *Sy*AcnB activity. The experiment was performed using 50 pmol of *Sy*AcnB. The red circles and triangles represent the specific activity for citrate at 45 °C. The blue circles and triangles represent the specific activity for isocitrate at 53 °C. Mean ± SD values were calculated from three independent experiments. (**b**) Effect of temperature on *Sy*AcnB activity. This was measured in Tris–HCl buffer pH 8.0. Mean ± SD values were calculated from three independent experiments. *Sy*AcnB, *Synechocystis* sp. PCC 6803 aconitase B; SD, standard deviation.

The activity of *Sy*AcnB for citrate was the highest at pH 7.7 and temperature 45–55 °C (Fig. 2a), and that for isocitrate as the substrate was the highest at pH 8.0 and a temperature of 53 °C (Fig. 2b). Thereafter, the activities of *Sy*AcnB for citrate were measured at pH 7.7 and 45 °C and for isocitrate at pH 8.0 and 53 °C except where indicated.

**Figure 3:**
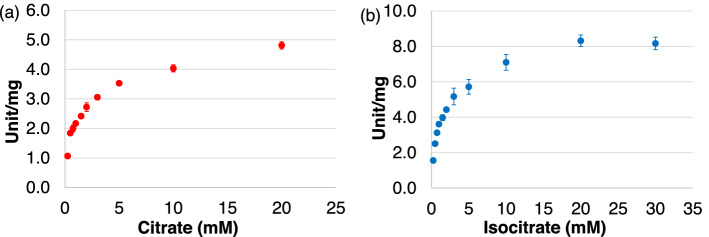
Saturation curves displaying the activities of *Sy*AcnB. The experiment was performed using (**a**) trisodium citrate dihydrate in Tris–HCl buffer pH 7.7 at 45 °C and (**b**) dl-isocitrate trisodium salt hydrate in Tris–HCl buffer pH 8.0 at 53 °C. Mean ± SD values were calculated from three independent experiments. *Sy*AcnB, *Synechocystis* sp. PCC 6803 aconitase B; SD, standard deviation.

The kinetic parameters of *Sy*AcnB, using citrate and isocitrate as the substrates, were estimated from the saturation curves (Fig. 3a,b). The *V*_max_, *k*_cat_, and *k*_cat_/*K*_m_ values of the activity of *Sy*AcnB for citrate were 4.58 ± 0.07 unit/mg, 9.12 ± 0.14 s^−1^, and 8.11 ± 0.23 s^−1^ mM^−1^, respectively (Table 2). The *V*_max_, *k*_cat_, and *k*_cat_/*K*_m_ values of the activity of *Sy*AcnB for isocitrate were 8.36 ± 0.17 unit/mg, 16.67 ± 0.34 s^−1^, and 10.88 ± 0.93 s^−1^ mM^−1^, respectively (Table 2). The *K*_m_ values of *Sy*AcnB for citrate and isocitrate were 1.13 ± 0.04 and 1.54 ± 0.17 mM, respectively (Table 3).

**Table 2:** Kinetic parameters of *Sy*AcnB.

Substrate	Condition	*V*_max_ (unit/mg)	*k*_cat_ (s^-1^)	*k*_cat_/*K*_m_ (s^-1^ mM^-1^)
Citrate	pH 7.7 at 45 °C (optimal)	4.58 ± 0.07	9.12 ± 0.14	8.11 ± 0.23
	pH 7.0	3.65 ± 0.19	7.28 ± 0.37	10.68 ± 0.76
	pH 8.0	3.51 ± 0.21	7.00 ± 0.41	8.79 ± 0.27
	pH 9.0	2.22 ± 0.09	4.42 ± 0.18	1.95 ± 0.22
Isocitrate	pH 8.0 at 53 °C (optimal)	8.36 ± 0.17	16.67 ± 0.34	10.88 ± 0.93
	pH 7.0	3.19 ± 0.18	6.36 ± 0.36	30.17 ± 1.51
	pH 8.0	4.52 ± 0.31	9.02 ± 0.61	19.13 ± 1.12
	pH 9.0	2.61 ± 0.11	5.19 ± 0.22	3.18 ± 0.47

The optimal conditions were measured for trisodium citrate dihydrate in Tris–HCl buffer pH 7.7 at 45 °C and for dl-isocitrate trisodium salt hydrate in Tris–HCl buffer pH 8.0 at 53 °C. The other six parameters were measured in Tris–HCl buffer pH 7.0, 8.0, and 9.0 at 30 °C. Mean ± standard deviation values were calculated from three independent experiments. The *P*-values between citrate and isocitrate calculated by Student’s *t*-test were listed in Table S3.

**Table 3:** List of *K*_m_ values with aconitase from various organisms.

Enzyme and organism	*K*_m_ (mM)		*K*_m_ ratio of Citrate/Isocitrate	References
	Citrate	Isocitrate		
*Synechocystis* sp. PCC 6803 (AcnB) under optimum conditions (Citrate: pH 7.7 at 45 °C, Isocitrate: pH 8.0 at 53 °C)	1.13	1.54	0.73	This study
*Corynebacterium glutamicum* (AcnA)	0.48	0.552	0.87	^30^
*Synechocystis* sp. PCC 6803 (AcnB) pH 9.0	2.28	1.65	1.38	This study
*Synechocystis* sp. PCC 6803 (AcnB) pH 8.0	0.80	0.47	1.70	This study
*Synechocystis* sp. PCC 6803 (AcnB) pH 7.0	0.68	0.21	3.24	This study
*Rattus norvegicus* (mitochondrial)	0.48	0.12	4.0	^35^
*Salmonella enterica* (AcnA)	5.3	0.9	5.89	^34^
*Sulfolobus acidocaldarius* (AcnA)	2.9	0.37	7.84	^29^
*Zea mays* (mitochondrial)	21.1	1.49	14.2	^36^
*Escherichia coli* (AcnA)	1.16	0.014	82.9	^25^
*Escherichia coli* (AcnB)	11	0.051	216	^25^

In *E. coli*, two different *K*_m_ values for isocitrate were obtained with different substrates at varying concentration ranges and compared with those measured for isocitrate (0.01–40 mM). The *P*-values between citrate and isocitrate calculated by Student’s *t*-test were listed in Table S3.

**Figure 4:**
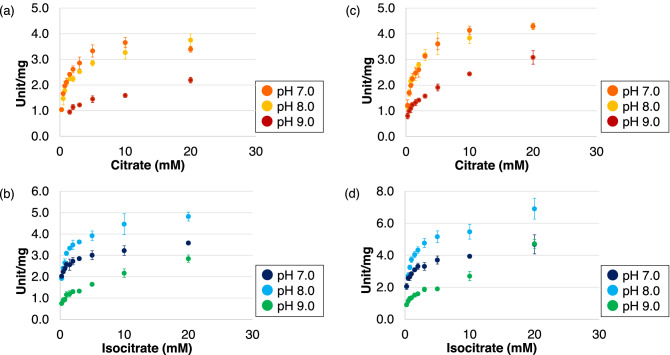
Saturation curves displaying the activities of *Sy*AcnB at each pH. The experiment was performed using (**a**) trisodium citrate dihydrate and (**b**) dl-isocitrate trisodium salt hydrate in Tris–HCl buffer pH 7.0, 8.0, and 9.0 at 30 °C. The experiment was repeated using (**c**) trisodium citrate dihydrate and (**d**) dl-isocitrate trisodium salt hydrate in Tris–HCl buffer pH 7.0, 8.0, and 9.0 at 45 °C. Mean ± SD values were calculated from three independent experiments. *Sy*AcnB, *Synechocystis* sp. PCC 6803 aconitase B; SD, standard deviation.

Kinetic parameters were calculated under optimal conditions for both substrates. Therefore, we calculated the parameters by unifying the measurement conditions and plotting a substrate saturation curve at 30 °C, which is the optimal temperature for the growth of *Synechocystis* 6803^23^ (Fig. 4a,b). In the presence of Tris–HCl (pH 7.0) at 30 °C, the *V*_*max*_ and *k*_cat_/*K*_m_ values of *Sy*AcnB for citrate were 3.65 ± 0.19 unit/mg and 10.68 ± 0.76 s^−1^ mM^−1^, respectively (Table 2). The *V*_*max*_ and *k*_cat_/*K*_m_ values for isocitrate were 3.19 ± 0.18 unit/mg and 30.17 ± 1.51 s^−1^ mM^−1^ respectively and 0.87- and 2.8-fold higher than those for citrate, respectively (Table 2). In the presence of Tris–HCl (pH 8.0) at 30 °C, the *V*_max_ and *k*_cat_/*K*_m_ values of *Sy*AcnB for citrate were 3.51 ± 0.21 unit/mg and 8.79 ± 0.27 s^−1^ mM^−1^, respectively (Table 2). The *V*_*max*_ and *k*_cat_/*K*_m_ values for isocitrate were 4.52 ± 0.31 unit/mg and 19.13 ± 1.12 s^−1^ mM^−1^ respectively and 1.3- and 2.2-fold higher than those for citrate, respectively (Table 2). Finally, in the presence of Tris–HCl (pH 9.0) at 30 °C, the *V*_max_ and *k*_cat_/*K*_m_ values of *Sy*AcnB for citrate were 2.22 ± 0.09 unit/mg and 1.95 ± 0.22 s^−1^ mM^−1^, respectively (Table 2). The *V*_*max*_ and *k*_cat_/*K*_m_ values for isocitrate were 2.61 ± 0.11 unit/mg and 3.18 ± 0.47 s^−1^ mM^−1^ respectively and 1.2- and 1.6-fold higher than those for citrate, respectively (Table 2). The *K*_m_ values of the activity of *Sy*AcnB for citrate at 30 °C were 0.68 ± 0.02 mM, 0.80 ± 0.03 mM, and 2.28 ± 0.16 mM at pH 7.0, 8.0, and 9.0, respectively, and the values for citrate were 3.2-, 1.7-, and 1.4-fold higher than those calculated for isocitrate at pH 7.0, 8.0, and 9.0, respectively (Table 3). Since there were some points where the correlation coefficient (R^2^ value) was low at 30 °C, the same measurement was performed at 45 °C (Fig. 4c,d). In the presence of Tris–HCl (pH 9.0) at 45 °C, the *V*_*max*_, *K*_m_ and *k*_cat_/*K*_m_ values of *Sy*AcnB for citrate were 2.88 ± 0.17 unit/mg, 1.58 ± 0.27 mM and 3.69 ± 0.61 s^−1^ mM^−1^, respectively, and the *V*_*max*_, *K*_m_ and *k*_cat_/*K*_m_ values for isocitrate were 4.64 ± 0.60 unit/mg, 3.79 ± 1.18 mM and 2.54 ± 0.50 s^−1^ mM^−1^ respectively (Table 4). All *K*_m_ values are summarised in Table S2. The results of adding the peptide AcnSP (aconitase small protein) showed that the *V*_max_, *K*_m_, and *k*_cat_/*K*_m_ values of *Sy*AcnB for citrate were 4.29 ± 0.08 unit/mg, 0.74 ± 0.09 mM, and 11.65 ± 1.24 s^−1^ mM^−1^, respectively, and the *V*_max_, *K*_m_, and *k*_cat_/*K*_m_ values of *Sy*AcnB for isocitrate were 6.59 ± 0.08 unit/mg, 0.91 ± 0.05 mM, and 14.50 ± 0.72 s^−1^ mM^−1^, respectively (Fig. S1). For both citrate and isocitrate, the addition of peptide AcnSP decreased the *V*_max_ and *K*_m_ values and increased the *k*_cat_/*K*_m_ value.

**Table 4:** Kinetic parameters of *Sy*AcnB.

Substrate	Condition	*V*_max_ (unit/mg)	*K*_m_ (mM^-1^)	*k*_cat_ (s^-1^)	*k*_cat_/*K*_m_ (s^-1^ mM^-1^)
Citrate	pH 7.0	4.35 ± 0.13	0.97 ± 0.12	8.67 ± 0.26	9.04 ± 0.82
	pH 8.0	4.21 ± 0.30	0.85 ± 0.17	8.40 ± 0.61	10.10 ± 1.44
	pH 9.0	2.88 ± 0.17	1.58 ± 0.27	5.74 ± 0.33	3.69 ± 0.61
Isocitrate	pH 7.0	4.09 ± 0.21	0.36 ± 0.05	8.16 ± 0.42	22.70 ± 2.19
	pH 8.0	6.25 ± 0.29	0.72 ± 0.11	12.45 ± 0.58	17.41 ± 1.90
	pH 9.0	4.64 ± 0.60	3.79 ± 1.18	9.25 ± 1.20	2.54 ± 0.50

These were measured in Tris–HCl buffer pH 7.0, 8.0, and 9.0 at 45 °C. Mean ± standard deviation values were calculated from three independent experiments. The *P*-values between citrate and isocitrate calculated by Student’s *t*-test were listed in Table S3.

### The activity of *Sy*AcnB in the presence of other TCA metabolites and cations

**Figure 5:**
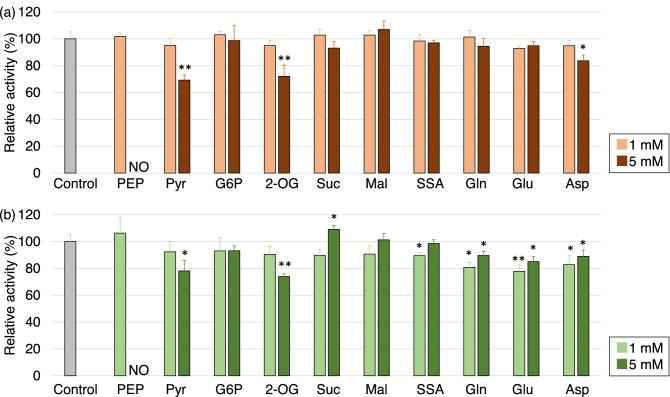
Effect of other TCA metabolites on the activity of *Sy*AcnB. The effect was analysed using (**a**) 1.13 mM trisodium citrate dihydrate and Tris–HCl buffer pH 7.7 at 45 °C and (**b**) 1.54 mM dl-isocitrate trisodium salt hydrate and Tris–HCl buffer pH 8.0 at 53 °C. Mean ± SD values were calculated from three independent experiments. The asterisks indicate significant differences compared to the *Sy*AcnB activities at the control condition (Student’s *t*-test; *P < 0.05, **P < 0.005). *Sy*AcnB, *Synechocystis* sp. PCC 6803 aconitase B; SD, standard deviation; NO, no enzymatic activity was detected; PEP, phosphoenolpyruvic acid monopotassium salt; Pyr, sodium pyruvate; G6P, glucose 6-phosphate; 2-OG, 2-oxoglutarate; Suc, disodium succinate; Mal, sodium l-malate; SSA, succinic semialdehyde; Gln, l-glutamine; Glu, l-glutamate; Asp, sodium l-aspartate monohydrate.

We examined the effects of various metabolites on *Sy*AcnB activity. The concentrations of the substrates used were the *K*_m_ values determined for each substrate. In the presence of 5 mM pyruvate, 2-OG, and l-aspartate, the activity of *Sy*AcnB for citrate decreased to 69%, 72%, and 84% of that of the control, respectively (Fig. 5a). Additionally, in the presence of 5 mM pyruvate, 2-OG, l-glutamine, l-glutamate, and l-aspartate, the activity of *Sy*AcnB for isocitrate decreased to 78%, 74%, 81%, 85%, and 89% of that of the control, respectively (Fig. 5b). The kinetic parameters of *Sy*AcnB in the absence (Table 2, 3) and the presence of 2-OG under optimal conditions (Fig. S2, S3) were compared. When citrate was used as a substrate, the addition of 1 mM 2-OG did not change the *V*_max_, *K*_m_, and *k*_cat_/*K*_m_ values (Fig. S2a), but the addition of 5 mM 2-OG increased the *K*_m_ value and decreased the *k*_cat_/*K*_m_ value (Fig. S3a). When isocitrate was used as a substrate, the addition of 1 mM 2-OG decreased the *V*_max_ and *K*_m_ values and increased *k*_cat_/*K*_m_ values (Fig. S2b), but the addition of 5 mM 2-OG decreased only the *V*_max_ value (Fig. S3b). As the parameters changed differently depending on the 2-OG concentration, the effect of 2-OG was studied by adding 0.44 mM 2-OG, similar to intracellular concentrations^24^, at 30 °C under three different pH conditions. The substrate concentrations were set to the *K*_m_ values listed in Table 2, respectively. There was no effect on the *Sy*AcnB activity irrespective of 2-OG presence at three pH conditions (Fig. S4).

**Figure 6:**
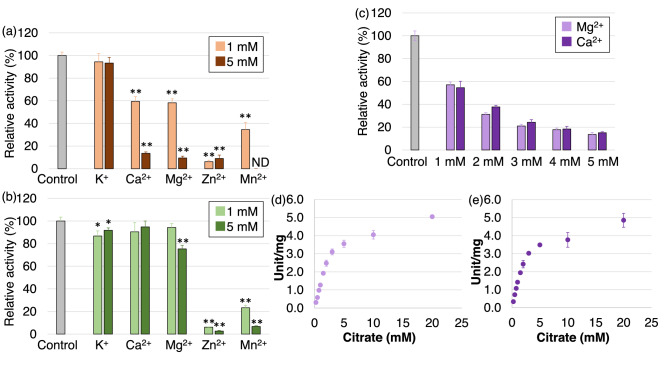
Effect of various cations on the activity of *Sy*AcnB. (**a**) The experiment was performed using (**a**) 1.13 mM trisodium citrate dihydrate in Tris–HCl buffer pH 7.7 at 45 °C and (**b**) 1.54 mM dl-isocitrate trisodium salt hydrate in Tris–HCl buffer pH 8.0 at 53 °C. The asterisks indicate significant differences compared to the *Sy*AcnB activities at the control condition (Student’s *t*-test; *P < 0.05, **P < 0.005). ND, enzymatic activity not determined; K, KCl; Ca, CaCl_2_; Mg, MgCl_2_·6H_2_O; Zn, ZnSO_4_·7H_2_O; Mn, MnCl_2_·4H_2_O. (**c**) The activities were measured using 1.13 mM trisodium citrate dihydrate and Tris–HCl buffer pH 7.7 at 45 °C in the presence of (**c**) 1–5 mM Mg^2+^ or Ca^2+^, (**d**) 1 mM Mg^2+^, and (**e**) 1 mM Ca^2+^. Mean ± SD values were calculated from three independent experiments. *Sy*AcnB, *Synechocystis* sp. PCC 6803 aconitase B; SD, standard deviation.

Furthermore, we examined the effects of monovalent and divalent cations on *Sy*AcnB activity. K^+^ had little effect on the activity of *Sy*AcnB for citrate, whereas the activity decreased to 59% and 14% in the presence of 1 mM and 5 mM Ca^2+^, respectively, and 58% and 9% in the presence of 1 mM and 5 mM Mg^2+^, respectively (Fig. 6a). Unlike the results obtained for citrate, 5 mM Mg^2+^ decreased the activity of *Sy*AcnB for isocitrate to 75%, and Ca^2+^ had little effect on the activity of *Sy*AcnB for isocitrate (Fig. 6b). The activity of *Sy*AcnB for citrate decreased to 6% and 35% in the presence of 1 mM Zn^2+^ and Mn^2+^, respectively, and 9% with 5 mM Zn^2+^ (Fig. 6a). Similarly, the activity of *Sy*AcnB for isocitrate decreased to 6% and 23% in the presence of 1 mM Zn^2+^ and Mn^2+^, respectively, and 3% and 7% in the presence of 5 mM Zn^2+^ and Mn^2+^, respectively (Fig. 6b).

We examined the effects of Mg^2+^ and Ca^2+^ on the kinetic parameters of the activity of *Sy*AcnB for citrate. The inhibitory effects of Mg^2+^ and Ca^2+^ on the activity of *Sy*AcnB for citrate were concentration-dependent (1–5 mM) (Fig. 6c). In the presence of 1 mM Mg^2+^, the *V*_max_ of the activity of *Sy*AcnB for citrate was 5.66 ± 0.20 unit/mg, and its *K*_m_ value increased to 3.01 ± 0.04 mM, whereas its *k*_cat_/*K*_m_ value decreased to 3.76 ± 0.12 s^−1^ mM^−1^ (Fig. 6d). Similarly, in the presence of 1 mM Ca^2+^, the *V*_max_ of the activity of *Sy*AcnB for citrate was 5.26 ± 0.49 unit/mg, and its *K*_m_ value increased to 2.61 ± 0.28 mM, whereas its *k*_cat_/*K*_m_ value decreased to 4.01 ± 0.10 s^−1^ mM^−1^ (Fig. 6e).

## Discussion

In this study, we demonstrated the biochemical properties of aconitase, which preferentially catalyses the reaction from isocitrate to citrate, in the unicellular cyanobacterium *Synechocystis* 6803 using citrate and isocitrate as the substrates.

We investigated the reactivation conditions for in vitro enzymatic reaction by altering the DTT concentration and incubation time. Previous studies have suggested the requirement of varying concentrations of DTT, such as 5 mM^22^ or 1 mM^25,26^, for aconitase reactivation. We also revealed that the maximum activity of the enzyme varied with DTT concentration. Additionally, various incubation times have been suggested for aconitase reactivation, for example, 20 min at 25 °C^26^ and 30–120 min on ice^22^. We demonstrated that aconitase was reactivated immediately after the addition of the reagents, and its maximum activity gradually decreased after 20 min. AcnB from *E. coli* is reactivated faster than AcnA, but the enzyme is unstable^10^. Thus, a long reactivation period for *Sy*AcnB may degrade the Fe-S cluster and reduce its activity.

The optimal pH required for *Corynebacterium glutamicum* aconitase (for citrate) is 7.5–7.8^26^ and that for *Mycobacterium tuberculosis* aconitase (for isocitrate) is 8.0^27^. These values are similar to the optimal pH values required for *Sy*AcnB activity in the presence of citrate and isocitrate (Fig. 2a). The intracellular pH of *Synechocystis* 6803 in logarithmically growing cells has been reported to be approximately 7.5–7.7 under dark conditions^28^. This suggests that the optimal pH required for *Sy*AcnB activity is suitable for the growth of *Synechocystis* 6803.

The optimal temperature required for *Sy*AcnB activity was estimated to be 45–55 °C (Fig. 2b), which is higher than the optimal growth temperature (30 °C) required for *Synechocystis* 6803, as reported in a previous study^23^. The optimal temperature required for the maximum activity of aconitase from *C. glutamicum* and the thermophilic archaea *Sulfolobus acidocaldarius* has been reported to be approximately 50 °C and 75 °C, respectively^26,29^. Additionally, the optimal temperature required for the maximum activity of aconitase from *C. glutamicum* is higher than its optimal growth temperature (30 °C)^30^. As the optimal temperature required for aconitase activity is known only for a few microorganisms, it remains unknown whether the optimal temperature for aconitase activity is usually higher than that required for the growth of microorganisms, as in this case. However, this pattern has been observed in some enzymes of the TCA cycle in *Synechocystis* 6803, such as fumarase (*Sy*Fum) (30 °C)^31^, wherein the optimal temperature required for enzyme activity corresponds with the optimal growth temperature of the bacterium; on the other hand, the optimal temperature required for the activity of other enzymes, such as citrate synthase (CS) from *Synechocystis* 6803 (*Sy*CS) (37 °C)^32^ and malate dehydrogenase (MDH) from *Synechocystis* 6803 (*Sy*MDH) (45–50 °C)^33^, is higher than the optimal growth temperature of the bacterium. Enzymes are thermally denatured and inactivated at high temperatures; however, the reaction rate increases with the increase in temperature. Therefore, the optimal temperature required for the activity of some enzymes may be higher than the optimal growth temperature of the microorganisms.

The affinity of *Sy*AcnB for citrate has been reported to be higher than that of aconitases from other microorganisms, namely, *E. coli*, *S. acidocaldarius*, and *Salmonella enterica* (Table 3)^25,29,34^. On the contrary, the affinity of *Sy*AcnB for isocitrate has been reported to be lower than that of aconitase from other microorganisms such as *E. coli*, *C. glutamicum*, *S. acidocaldarius*, and *S. enterica* (Table 3)^25,26,29,34^. The *K*_m_ value of the activity of aconitase from *C. glutamicum* for citrate was slightly lower than that for isocitrate, which is consistent with the results obtained for *Sy*AcnB, whereas the *K*_m_ values of the activity of aconitase from *E. coli*, *S. acidocaldarius*, *S. enterica*, *Rattus norvegicus* (mitochondrial), and *Zea mays* (mitochondrial) for citrate are higher than those reported for isocitrate (Table 3)^25,26,29,34–36^. The calculated values for *K*_m_ (citrate)/*K*_m_ (isocitrate) ratio are shown in Table 3; the ratio was estimated to be 0.73 at the optimum activity of *Sy*AcnB, which is close to that for *C. glutamicum* aconitase (0.87). At pH 7.0, 8.0, and 9.0, the ratios were above 1 but were lower than those estimated for other organisms, except for *C. glutamicum* (Table 3). These results suggest that the aforementioned microorganisms tend to oxidise citrate to isocitrate. These values correspond with higher intracellular concentrations of citrate than isocitrate in *Synechocystis* 6803, which was estimated by the absolute quantification of metabolites^17^. The *k*_cat_/*K*_m_ value of the activity of *Sy*AcnB for isocitrate was slightly higher than that for citrate; this value is similar to that for aconitase from *C. glutamicum* (40.8 s^−1^ mM^−1^ for citrate and 52.4 s^−1^ mM^−1^ for isocitrate) and *S. enterica* (1.00 s^−1^ mM^−1^ for citrate and 1.22 s^−1^ mM^−1^ for isocitrate)^26,34^. Unlike heterotrophic bacteria, the TCA cycle flux in *Synechocystis* 6803 is always low under photoautotrophic, photomixotrophic, and heterotrophic conditions^37–40^, which may explain why the *k*_cat_/*K*_m_ value of *Sy*AcnB is lower than that of *C. glutamicum* aconitase.

The higher the pH, the lower the *K*_m_ (citrate)/*K*_m_ (isocitrate) ratio of *Sy*AcnB (Table 3). The direction of the TCA cycle in *Synechocystis* 6803 is strongly affected by the pH, and the in vitro reconstruction of oxaloacetate metabolism displays a higher yield of citrate at higher pH^41^. At higher pH, higher concentrations of citrate, which is the substrate for *Sy*AcnB, are formed, and the reaction is more likely to proceed in the oxidative direction at chemical equilibrium.

The TCA cycle in *Synechocystis* 6803 is characterised by the citrate accumulation at high levels in the cells, although 2-OG is generated through the oxidative TCA cycle from citrate under normal phototrophic growth conditions^17^. In *Synechocystis* 6803, isocitrate dehydrogenase (ICD) can catalyse isocitrate to form 2-OG. The *K*_m_ value of the activity of ICD from *Synechocystis* 6803 (*Sy*ICD) for isocitrate was estimated to be 5.7 × 10^–3^–5.9 × 10^–2^ mM^42^. The *K*_m_ values of the activity of *Sy*AcnB for isocitrate were 3.6-fold higher than those of the activity of *Sy*ICD. Therefore, isocitrate is thought to be metabolised mainly by *Sy*ICD, rather than by *Sy*AcnB, enabling the cells to produce 2-OG. The three lines of evidence, 1) the high level of citrate accumulation in *Synechocystis* 6803, 2) the lack of citrate-metabolising enzymes such as ACL and CCS/CCL^43–45^ (Table 1), and 3) the lack of *Sy*CS activity degrading a citrate^32^, suggest that *Sy*AcnB enhances the reaction in the direction of citrate to isocitrate to produce 2-OG. In this way, the properties of two enzymes, *Sy*AcnB and *Sy*ICD, facilitate citrate accumulation and 2-OG generation at the same time. The peptide AcnSP affects the kinetic parameters of *Sy*AcnB^21^ and we performed biochemical analysis using AcnSP (Fig. S1). In both cases, *V*_max_ and *K*_m_ values decreased as in previous studies using *cis*-aconitate as a substrate, suggesting that AcnSP does not a significant effect on the reaction direction but boosting the reaction between citrate and isocitrate catalysed by *Sy*AcnB.

*Sy*AcnB activities in both directions were inhibited by 2-OG in our study (Fig. 5); however, 2-OG has not been reported as an inhibitor of aconitase thus far. Therefore, we tested the effects of 2-OG in detail by comparing the kinetic parameters when 1 or 5 mM 2-OG was added (Fig. S2, S3) with those when it was not added (Table 2). When citrate was used as a substrate, 5 mM 2-OG acts as an inhibitor (Fig. S3a), but not at 1 mM 2-OG deduced from *k*_cat_/*K*_m_ values of *Sy*AcnB (Fig. S2a). Whereas when isocitrate was used as a substrate, 1 mM 2-OG acts as an activator (Fig. S2b), but not at 5 mM 2-OG deduced from *k*_cat_/*K*_m_ values of *Sy*AcnB (Fig. S3b). The addition of 0.44 mM 2-OG, the intracellular concentration in *Synechocystis* cells^24^, did not decrease *Sy*AcnB activities (Fig. S4), and hence, 2-OG could play a role in the inhibition of *Sy*AcnB activities when too many reactions of the oxidative reaction of the TCA cycle have proceeded. We also found that the activity of aconitase from *Z. mays* (mitochondrial) is inhibited by succinate and malate^36^, whereas that of *Sy*AcnB was not inhibited by these organic acids.

The activity of *Sy*AcnB for citrate was strongly inhibited by Mg^2+^ and Ca^2+^ ions (Fig. 6a). Mg^2+^ and Ca^2+^ increased the *K*_m_ value. The *k*_cat_/*K*_m_ values for citrate in the presence of 1 mM Mg^2+^ and Ca^2+^ were estimated to be 46% and 49% of the control, respectively. As per a previous report, the citrate/isocitrate concentration ratio for aconitase from rat heart was altered by Mg^2+^ and Ca^2+^ ions, and the equilibrium leaned towards citrate^46^. Comparing the effects of Mg^2+^ and Ca^2+^ on the activities of enzymes in the TCA cycle from *Synechocystis* 6803 revealed that the activity of *Sy*CS increased to 1463% and 1050% of the control in the presence of 100 mM Mg^2+^ and Ca^2+^, respectively, and that the activity of *Sy*MDH increased to 160% and 190% of the control in the presence of 1 mM and 10 mM Mg^2+^, respectively^32,33^. Additionally, *Sy*ICD requires Mg^2+^ or Mn^2+^ as a cofactor for its activity^42^. The concentration of free Mg^2+^ ions in the stroma of spinach chloroplasts varies between dark and light conditions^47^. Thus, depending on culture conditions, the concentration of free Mg^2+^ in *Synechocystis* 6803 cells may be altered^48^, which may affect the equilibrium of aconitase. Also, *Sy*AcnB activities in both directions were strongly inhibited by Mn^2+^ and Zn^2+^ (Fig. 6a,b). The mitochondrial aconitase activity from rat AF5 cells decreased to 48% and 19% of the control in the presence of 2 mM and 5 mM Mn^2+^, respectively^49^. The activity of aconitase from rat prostate epithelial cells for citrate was inhibited by Zn^2+^, but this effect was not observed for isocitrate^50^. The activity of *Sy*CS decreased to 37% of that in the control in the presence of 100 mM Mn^2+^^32^, and the activity of *Sy*Fum was inhibited by 10 mM Mn^2+^ when l-malate was used as a substrate^31^. Moreover, the activity of *Sy*Fum was strongly inhibited by 1 mM Zn^2+^^31^. Presently, the understanding of the physiological significance of metal ions in *Synechocystis* 6803 is limited.

In this study, we determined the biochemical properties of *Sy*AcnB and demonstrated that citrate accumulation depends on the enzyme kinetics of *Sy*AcnB. The consumption of isocitrate by *Sy*ICD to produce 2-OG overcomes the kinetic barrier of the *Sy*AcnB enzyme. Currently, the study is limited to biochemical analysis; further genetic manipulation of *Sy*AcnB might reveal its importance in citrate metabolism in cyanobacteria.

## Methods

### Construction of cloning vector for the expression of recombinant *Sy*AcnB

The nucleotide sequence of *acnB* (slr0665), obtained from the sequenced genome of *Synechocystis* 6803 at KEGG database (https://www.genome.jp/kegg/kegg_ja.html), was synthesised by Eurofins Genomics Japan (Tokyo, Japan). The synthesised fragment was inserted within the *Bam*HI*–Xho*I site of the vector pGEX6P-1 (GE Healthcare Japan, Tokyo, Japan).

The cloned expression vector was transformed in competent *E. coli* DH5α cells (Takara Bio, Shiga, Japan), and the transformed *E. coli* cells were cultivated in 5 L of Luria–Bertani medium at 30 °C with shaking at 150 rpm. Recombinant protein expression was induced overnight by adding 0.01 mM isopropyl β-D-1-thiogalactopyranoside (Wako Chemicals, Osaka, Japan) to the medium.

### Affinity purification of the recombinant protein

The recombinant *E. coli* DH5α cells from 800 mL culture were suspended in 40 mL of phosphate-buffered saline/tween (PBST) (1.37 M NaCl, 27 mM KCl, 81 mM Na_2_HPO_4_·12H_2_O, 14.7 mM KH_2_PO_4_, and 0.05% Tween 20) and lysed through sonication (model VC-750; EYELA, Tokyo, Japan). The procedure was repeated 10 times for 10 s at 20% intensity. The lysed cells were centrifuged at 13,000 × *g* for 15 min at 4 °C. The supernatant was transferred to a 50-mL tube, and 560 µL of Glutathione Sepharose 4 B resin (GE Healthcare Japan, Tokyo, Japan) was added. Thereafter, the mixture was gently shaken for 30 min on ice. To remove the supernatant, the mixture was centrifuged at 5,800 × *g* for 2 min at 4 °C. The resin was re-suspended in 700 µL of PBST and washed five times. After washing, the recombinant protein was eluted with 700 µL of glutathione-*S*-transferase (GST) elution buffer (50 mM Tris–HCl (pH 9.6) and 10 mM reduced glutathione) five times, and the protein was concentrated using a Vivaspin 500 MWCO 50,000 device (Sartorius, Göttingen, Germany). The protein concentration was measured using a Pierce BCA Protein Assay Kit (Thermo Fisher Scientific, Rockford, IL, USA). To verify protein purification, sodium dodecyl sulphate–polyacrylamide gel electrophoresis was carried out, and the gel was stained using Instant Blue reagent (Expedeon Protein Solutions, San Diego, CA, USA).

### Enzyme assay

Before measuring the enzyme activity, purified 50 pmol *Sy*AcnB was reactivated by adding 25 µL of a solution containing 5 mM dl-dithiothreitol (DTT), 100 µM Na_2_S, and 100 µM (NH_4_)_2_Fe(SO_4_)_2·_6H_2_O and incubating the mixture at 20 °C for 1 ~ 30 min. The activity of *Sy*AcnB was measured by mixing 50 pmol holo-*Sy*AcnB with 1 mL of the assay solution (100 mM Tris–HCl (pH 7.0–9.0) or MES-NaOH (pH 6.0–7.0) and 20 mM trisodium citrate dihydrate or 20 mM dl-isocitrate trisodium salt hydrate). The enzymatic reaction was initiated by adding reactivated *Sy*AcnB. The formation of *cis*-aconitate was monitored by measuring the absorbance at 240 nm using a Hitachi U-3310 spectrophotometer (Hitachi High-Tech, Tokyo, Japan)^51^. One unit of *Sy*AcnB activity was defined as the formation of 1 µmol *cis*-aconitate per minute. Unit/mg represents the value of one unit divided by the amount of purified protein (mg). The *K*_m_ and *V*_max_ values were calculated using curve fitting of Michaelis–Menten equation with the KaleidaGraph ver. 4.5 software and the *k*_cat_ values were calculated from *V*_max_ values. The 44 amino acid sequence of AcnSP from *Synechocystis* 6803 was synthesized by Eurofins Genomics Japan (Tokyo, Japan) with a purity of 91.6%.

### Statistical analysis

Paired two-tailed Student's *t*-tests were performed to calculate the *P*-values using Microsoft Excel for Windows (Redmond, WA, USA). All experiments were independently carried out three times.

## Supplementary Information

Supplementary Information.

## Data Availability

All the materials and data are available by contacting the corresponding author.
