# Supplementary material for: Biochemical elucidation of citrate accumulation in Synechocystis sp. PCC 6803 via kinetic analysis of aconitase
Source: Sci Rep. 2021 Aug 24;11:17131. doi: 10.1038/s41598-021-96432-2 (PMC8385029; doi:10.1038/s41598-021-96432-2)
Supplement: Supplementary file 1 — Supplementary Information. [file 41598_2021_96432_MOESM1_ESM.docx]

**Figure S1. Saturation curves for the calculation of the activities of *Sy*AcnB in the presence of 50 pmol AcnSP.** The experiment was performed using (a) trisodium citrate dihydrate in Tris-HCl buffer pH 7.7 at 45℃ and (b) dl-isocitrate trisodium salt hydrate in Tris-HCl buffer pH 8.0 at 53℃ by adding 50 pmol of AcnSP to the reaction mixture. Mean ± SD values were calculated from three independent experiments. *Sy*AcnB, *Synechocystis* sp. PCC 6803 aconitase B; SD, standard deviation. The asterisks indicate that significant differences between the absence and presence of AcnSP under optimal conditions (Student’s *t*-test; **P* < 0.05, ***P* < 0.005). The *P*-values calculated by Student’s *t*-test were listed in Table S4.

**
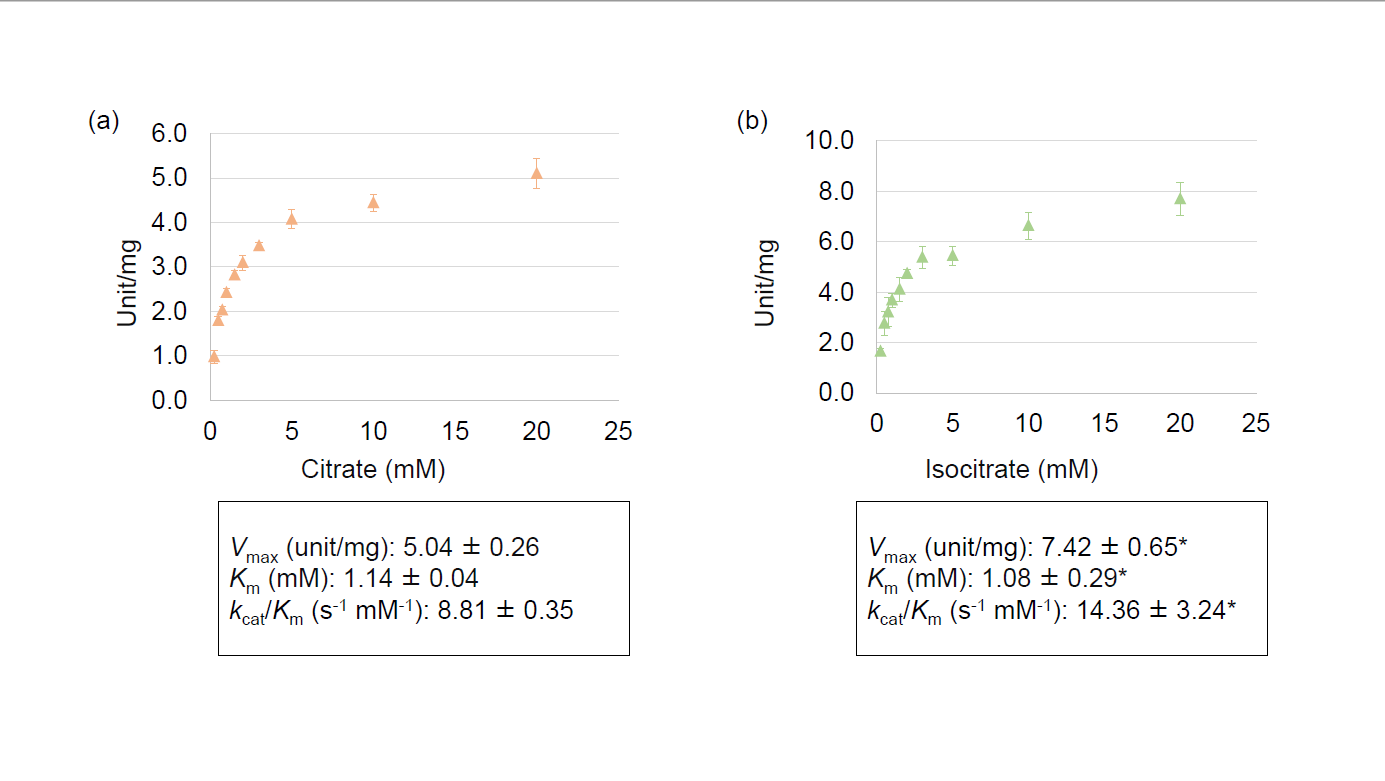
**

**Figure S2. Saturation curves for the calculation of the activities of *Sy*AcnB in the presence of 1 mM 2-OG.** The experiment was performed using (a) trisodium citrate dihydrate in Tris-HCl buffer pH 7.7 at 45℃ and (b) dl-isocitrate trisodium salt hydrate in Tris-HCl buffer pH 8.0 at 53℃ by adding 1 mM 2-OG to the reaction mixture. Mean ± SD values were calculated from three independent experiments. *Sy*AcnB, *Synechocystis* sp. PCC 6803 aconitase B; SD, standard deviation. The asterisks indicate that significant differences between the absence and presence of 1 mM 2-OG under optimal conditions (Student’s *t*-test; **P* < 0.05, ***P* < 0.005). The *P*-values calculated by Student’s *t*-test were listed in Table S4.

**Figure S3. Saturation curves for the calculation of the activities of *Sy*AcnB in the presence of 5 mM 2-OG.** The experiment was performed using (a) trisodium citrate dihydrate in Tris-HCl buffer pH 7.7 at 45℃ and (b) dl-isocitrate trisodium salt hydrate in Tris-HCl buffer pH 8.0 at 53℃ by adding 5 mM 2-OG to the reaction mixture. Mean ± SD values were calculated from three to six independent experiments. *Sy*AcnB, *Synechocystis* sp. PCC 6803 aconitase B; SD, standard deviation. The asterisks indicate that significant differences between the absence and presence of 5 mM 2-OG under optimal conditions (Student’s *t*-test; **P* < 0.05, ***P* < 0.005). The *P*-values calculated by Student’s *t*-test were listed in Table S4.

**Figure S4.** **Effect of intracellular 2-OG concentration on the activity of *Sy*AcnB at each pH.** The experiment was performed using (a) trisodium citrate dihydrate and (b) dl-isocitrate trisodium salt hydrate in Tris-HCl buffer pH 7.0, 8.0, and 9.0 at 30℃ by adding 0.44 mM 2-OG to the reaction mixture. Substrate concentrations are the *K*_m_ values for each condition ((a) pH 7.0: 0.68 mM, pH 8.0: 0.80 mM, pH 9.0: 2.28 mM, (b) pH 7.0: 0.21 mM, pH 8.0: 0.47 mM, pH 9.0: 1.65 mM). Mean ± SD values were calculated from three independent experiments. *Sy*AcnB, *Synechocystis* sp. PCC 6803 aconitase B; SD, standard deviation.

**Table S1. BLAST search results for aconitaseA**

| Query sequence | NCBI-ProteinID | Name | K number | Bits | E-value |
| --- | --- | --- | --- | --- | --- |
| A | NP_460671 | acnA; aconitate hydratase 1 | K01681 | 1695 | 0.0 |
|  | NP_215991 | acn; iron-regulated aconitate hydratase | K01681 | 1009 | 0.0 |
|  | NP_600755 | Cgl1540; aconitate hydratase | K01681 | 1001 | 0.0 |
|  | AAY80560 | acnA; aconitate hydratase | K01681 | 856 | 0.0 |
|  | BAA18738 | leuC; 3-isopropylmalate dehydratase | K01703 | 106 | 5e-25 |
|  | BAA18298 | leuD; 3-isopropylmalate dehydratase | K01704 | 37.7 | 0.002 |
|  | BAA18689 | hypothetical protein | K01682 | 35.0 | 0.024 |
|  | BAA17569 | hypothetical protein |  | 32.0 | 0.16 |
|  | BAA18190 | ribA; GTP cyclohydrolase II | K14652 | 28.9 | 1.4 |
|  | BAA17576 | unknown protein |  | 27.3 | 3.2 |
|  | BAA18257 | melB; melibiose carrier protein | K03292 | 27.3 | 4.7 |
|  | BAA17789 | rimI; ribosomal-protein-alanine acetyltransferase | K03789 | 26.2 | 5.5 |
|  | BAA18131 | int; apolipoprotein N-acyltransferase | K03820 | 26.2 | 9.6 |

BLAST search for aconitaseA was performed using the Kyoto Encyclopedia of Genes and Genomes database (<https://www.genome.jp/kegg/genome.html>). The following sequences were used for the search: A: aconitate hydratase 1 from *Escherichia coli* K-12 W3110 (JW1268).

**Table S2. List of all *K*_m_ values with aconitase from various organisms**

| Enzyme and organism | *K*_m_ (mM) |  | *K*_m_ ratio of Citrate/Isocitrate | Reference |
| --- | --- | --- | --- | --- |
|  | Citrate | Isocitrate |  |  |
| *Synechocystis* sp. PCC 6803 (AcnB) pH 9.0 (45℃) | 1.58 | 3.79 | 0.42 | this study |
| *Synechocystis* sp. PCC 6803 (AcnB) under optimum conditions (Citrate: pH 7.7 at 45℃, Isocitrate: pH 8.0 at　53℃) | 1.13 | 1.54 | 0.73 | this study |
| *Corynebacterium glutamicum*  (AcnA) | 0.48 | 0.552 | 0.87 | [30] |
| *Synechocystis* sp. PCC 6803 (AcnB) pH 8.0 (45℃) | 0.85 | 0.72 | 1.18 | this study |
| *Synechocystis* sp. PCC 6803 (AcnB) pH 9.0 (30℃) | 2.28 | 1.65 | 1.38 | this study |
| *Synechocystis* sp. PCC 6803 (AcnB) pH 8.0 (30℃) | 0.80 | 0.47 | 1.70 | this study |
| *Synechocystis* sp. PCC 6803 (AcnB) pH 7.0 (45℃) | 0.97 | 0.36 | 2.69 | this study |
| *Synechocystis* sp. PCC 6803 (AcnB) pH 7.0 (30℃) | 0.68 | 0.21 | 3.24 | this study |
| *Rattus norvegicus* (mitochondrial) | 0.48 | 0.12 | 4.0 | [35] |
| *Salmonella enterica* (AcnA) | 5.3 | 0.9 | 5.89 | [34] |
| *Sulfolobus acidocaldarius*  (AcnA) | 2.9 | 0.37 | 7.84 | [29] |
| *Zea mays* (mitochondrial) | 21.1 | 1.49 | 14.2 | [36] |
| *Escherichia coli* (AcnA) | 1.16 | 0.014 | 82.9 | [25] |
| *Escherichia coli* (AcnB) | 11 | 0.051 | 216 | [25] |

In *E. coli*, two different *K*_m_ values for isocitrate were obtained with different substrates at varying concentration ranges and compared with those measured for isocitrate (0.01–40 mM).

**Table S3. List of *P*-values between citrate and isocitrate calculated by Student’s *t*-test in Table 2**–**4**

| Conditions | *V*_max_ | *K*_m_ | *k*_cat_/*K*_m_ |
| --- | --- | --- | --- |
| optimal | 0.0001 | 0.0427 | 0.0297 |
| pH 7.0 at 30℃ | 0.0370 | 0.0000 | 0.0004 |
| pH 8.0 at 30℃ | 0.0129 | 0.0043 | 0.0026 |
| pH 9.0 at 30℃ | 0.0101 | 0.0105 | 0.0281 |
| pH 7.0 at 45℃ | 0.1604 | 0.0057 | 0.0040 |
| pH 8.0 at 45℃ | 0.0011 | 0.3623 | 0.0074 |
| pH 9.0 at 45℃ | 0.0295 | 0.0774 | 0.0684 |

The *P*-values comparing citrate and isocitrate were calculated from Table 2-4.

**Table S4. List of *P*-values calculated by Student’s *t*-test in Figure S1–S3**

| Substrate | Effector | *V*_max_ | *K*_m_ | *k*_cat_/*K*_m_ |
| --- | --- | --- | --- | --- |
| Citrate | AcnSP  (Fig. S1a) | 0.0106 | 0.0084 | 0.0349 |
| Isocitrate | AcnSP  (Fig. S1b) | 0.0007 | 0.0170 | 0.0070 |
| Citrate | 1 mM 2-OG  (Fig. S2a) | 0.0827 | 0.6028 | 0.0566 |
| Citrate | 5 mM 2-OG  (Fig. S3a) | 0.8264 | 0.0042 | 0.0021 |
| Isocitrate | 1 mM 2-OG  (Fig. S2b) | 0.0151 | 0.0217 | 0.0479 |
| Isocitrate | 5 mM 2-OG  (Fig. S3b) | 0.0010 | 0.2004 | 0.2568 |

The *P*-values were calculated from the optimum conditions in Tables 2, 3 and Fig. S1–S3.
